# Scientific Items

**Published:** 1888-07

**Authors:** 


### SCIENTIFIC ITEMS.

**The Growth of Science.**—From primitive times man knew where the sun would rise on a certain day; and the record of this knowledge is left us in the prehistoric buildings, if such they may be called, of Britain. At Greenwich the moon has been observed, with scarcely an intermission, for a hundred and fifty years, but not for the purpose of seeing what it was made of, but for the purpose of forming the lunar tables, by means of the moon's place among the stars, will give the navigator his positions. In the same way at the Washington observatory one may see a wonderfully exact instrument strongly bolted to massive piers, and so immovable that the sun can be observed by it but once daily as it crosses the meridian. This instrument is the complete attainment of that long line of progress in one direction of which the prehistoric stones at Stonehenge mark the initial step—the attainment, that is, purely of precision of measurement. The new branch of astronomy, which has had its entire growth within a few years, studies sun, moon, and stars for what they are in themselves and in relation to ourselves. Its study of the sun, beginning with its external features, led to the further inquiry as to what it was made of, and then to finding the unexpected relations which it bore to the earth and our own daily lives on it. This new branch of inquiry is what Professor Langley calls the "new astronomy"—this study of the celestial bodies to find out their nature and their relation to us, rather than for the purpose of simply recording their relative motions—that Professor Langley has made so beautiful and so eloquent an appeal for the proper endowment of this new field of research. No one can read this book of Langley's without feeling that astronomy has acquired an entirely new interest for him. It now results in something more than the dry-looking pages upon pages of tables.—*Science.*

**Magazine Guns.**—The repeating rifle of the German army differs from the ordinary rifle in the fact that the stock, instead of stopping short where it is grasped by the left hand, is prolonged to within an inch of the end of the barrel. This constitutes the reservoir of cartridges. The firing consists of three movements—the "ready," during which each man gives a sharp turn to the right to a lever above the lock of his gun, and the familiar "present" and "fire." The company stand four deep, the two front ranks firing while the two rear ranks recharge their magazines. So rapid are the movements that the magazine is emptied, with a perceptible allowance each time for rapid aim, in ten seconds. To think of what would happen to any body of men exposed to half a minute of firing like this is simply appalling.

It is all very well for a Russian governor to issue orders to his troops, calling upon them to remember that battles are won by courage, and not by repeating rifles; but the moral effect of the new weapon both upon those using it and those opposed to it must be enormous. To be able to wait until the enemy, in whatever form he may be, is within a few yards of you, and then deliver your fire upon him at the rate of a shot a second, is quite enough to revolutionize all attack formations.—*Broad Arrow.*

**The Great Yellowstone Geyser Now Active.**—A dispatch to the Chicago *Tribune* says the Excelsior geyser in the Yellowstone Park is in operation. This geyser is in the great middle geyser basin, close to Fire Hole River. It is in the form of an immense pit 320 feet in length and 200 feet wide, and the aperture through which it discharges its volume of water is nearly 200 feet in diameter. Its general appearance is that of a huge boiling spring, and for many years its true character was not suspected. Its first eruption occurred in 1880, when it revealed itself as a stupendous geyser. The power of its eruptions was almost incredible, sending an immense column of water to heights of from 100 to 300 feet, and hurling with it rocks and bowlders of from 1 to 100 pounds in weight. Its present eruption is said to be a repetition of that of 1880. It is throwing its volumes of water 300 feet into the air, and Fire Hole River is reported to have risen two feet from its rushing floods. This is now conceded to be the most powerful geyser in existence.—*Scientific American.*

**The** way in which glass may best be cut with scissors is told in the *Pottery Gazette*, London: Glass may be cut under water with great ease, to almost any shape, with a pair of shears or strong scisors. Two things are necessary for success. First, the glass must be kept quite level in the water while the scissors are applied; and secondly, to avoid risk, it is better to perform the cutting by taking off small pieces at the corners and along the edges, and to reduce the shape gradually to that required. The softer glasses cut the best, and the scissors need not be very sharp.—*Ib.*

**The Edison Photophone.**—The Editor of the *Western Electrician* thinks the Edison photophone possesses such vast possibilities and its achievement has awakened an enthusiasm which has not been manifest since the introduction of the telephone. It may, he thinks, serve a thousand different purposes. It may aid the business man throughout the working hours and charm him in his leisure moments. Employed as it can be for both pleasure and business, it may revolutionize life in both these aspects.—*Ib.*

**The Sun as an Incendiary.**—The *Chemist and Druggist* (London) records the fact of a chemist shop just opened, at 16 High Street, with show bottles in the windows, which, acting as burning glass, set fire to the store. It was discovered before much damage was done, but serves as another warning against placing show bottles where the sun can reach them in show windows.—*Scientific American.*

**The area of dry land** of the world is estimated at 55,000,000 square miles, the area of the ocean 137,200,000 square miles. The bulk of the dry land above the level of the sea is 23,450,000 cubic miles, and the volumes of the waters of the ocean is 323,800,000 miles. The mean height of the land is 2,250 feet. The mean depth of the whole ocean is 12,480 feet.—*Phrenological Journal.*

